# Response assessment of GBM during immunotherapy by delayed contrast treatment response assessment maps

**DOI:** 10.3389/fneur.2024.1374737

**Published:** 2024-04-08

**Authors:** Valeria Cuccarini, Filippo Savoldi, Yael Mardor, David Last, Serena Pellegatta, Federica Mazzi, Maria Grazia Bruzzone, Elena Anghileri, Bianca Pollo, Luisa Maddaloni, Camilla Russo, Elisa Bocchi, Valentina Pinzi, Marica Eoli, Domenico Aquino

**Affiliations:** ^1^Neuroradiology Unit, Fondazione IRCCS Istituto Neurologico Carlo Besta, Milan, Italy; ^2^Advanced Technology Center, Sheba Medical Center, Ramat Gan, Israel; ^3^Tel Aviv University, Tel Aviv, Israel; ^4^Molecular Neuro-Oncology Unit, Fondazione IRCCS Istituto Neurologico Carlo Besta, Milan, Italy; ^5^Neuro-Pathology Unit, Fondazione IRCCS Istituto Neurologico Carlo Besta, Milan, Italy; ^6^Dipartimento di Ingegneria Elettrica e delle Tecnologie dell’Informazione (DIETI), Università Degli Studi di Napoli “Federico II”, Naples, Italy; ^7^Radiotherapy Unit, Fondazione IRCCS Istituto Neurologico Carlo Besta, Milan, Italy

**Keywords:** glioblastoma, immunotherapy, Stupp protocol, magnetic resonance imaging, pseudoprogression, TRAMs, immunological responders, natural killer cells

## Abstract

**Introduction:**

Assessing the treatment response of glioblastoma multiforme during immunotherapy (IT) is an open issue. Treatment response assessment maps (TRAMs) might help distinguish true tumor progression (TTP) and pseudoprogression (PsP) in this setting.

**Methods:**

We recruited 16 naïve glioblastoma patients enrolled in a phase II trial consisting of the Stupp protocol (a standardized treatment for glioblastoma involving combined radiotherapy and chemotherapy with temozolomide, followed by adjuvant temozolomide) plus IT with dendritic cells. Patients were followed up till progression or death; seven underwent a second surgery for suspected progression. Clinical, immunological, and MRI data were collected from all patients and histology in case of second surgery. Patients were classified as responders (progression-free survival, PFS > 12 months), and non-responders (PFS ≤ 12), HIGH-NK (natural killer cells, i.e., immunological responders), and LOW-NK (immunological non-responders) based on immune cell counts in peripheral blood. TRAMs differentiate contrast-enhancing lesions with different washout dynamics into hypothesized tumoral (conventionally blue-colored) vs. treatment-related (red-colored).

**Results:**

Using receiver operating characteristic (ROC) curves, a threshold of −0.066 in V_Blue/_V_CE_ (volume of the blue portion of tumoral area/volume of contrast enhancement) variation between values obtained in the MRI performed before PsP/TTP and at TTP/PSP allowed to discriminate TTP from PsP with a sensitivity of 71.4% and a specificity of 100%. Among HIGH-NK patients, at month 6 there was a significant reduction compared to baseline and month 2 in median “blue” volumes.

**Discussion:**

In conclusion, in our pilot study TRAMs support the discrimination between tumoral and treatment-related enhancing features in immunological responders vs. non-responders, the distinction between PsP and TTP, and might provide surrogate markers of immunological response.

## 1 Introduction

Glioblastoma multiforme (GBM) is the most frequent malignant primary brain tumor; it mainly affects adults and carries an ominous prognosis (1). The current therapy standard of care (SOC) consists of maximal safe surgical resection followed by the Stupp protocol (2), i.e., radiation therapy and adjuvant temozolomide (TMZ), with a median overall survival (OS) of 14–16 months. Recurrence, almost inevitable due to GBM infiltrative behavior, has no accepted standard treatment. The recent successful application of immunotherapy (IT) in the care of melanoma, lung cancer, and renal cancer (3) and the revolutionary discovery of a glymphatic system inside the central nervous system (CNS) (4) have awakened the interest in IT in malignant brain tumors. At present, more than 20% of the ongoing clinical trials in GBM are exploiting immunotherapeutic interventions. Immune checkpoint inhibitors (immune modulators), used also for extra-cerebral tumors, have been first tested with limited survival gain benefits (5), likely in relation to the ability of GBM to escape immune surveillance by various mechanisms (6–10). Vaccine-based active immunotherapies can produce a stronger immune stimulation and might overcome this limitation (11). In particular, vaccination with dendritic cells (DC) loaded with tumor peptides has shown good safety profiles and increased OS in clinical trials (11–13). Two clinical studies, DENDR1 (NCT04801147) and DENDR2 (NCT04002804), including, respectively, the treatment of first diagnosis and recurrent GBM patients with DCs loaded with autologous tumor lysate, were activated at the Fond. IRCCS Istituto Neurologico C. Besta. The DENDR2 study was stopped due to lack of clear efficacy (14); on the contrary, the DENDR1 study is still active. In this latter study, we observed that DC-IT was capable of inducing an anti-tumor immune response. The increased survival observed in responders was associated with long-lasting natural killers (NK), but not with the response of CD8+ T lymphocytes (12).

Standard treatment response assessment in gliomas relies on MRI. A transient increase in enhancing volume has been described in up to 20–30% of patients after the Stupp protocol (15–17): This inflammatory-based pseudoprogression (PsP) eventually subsides and should be distinguished from true tumor progression (TTP) to avoid early discontinuation of effective treatments. This problem is magnified with IT, which induces a stronger inflammatory response. Response Assessment for Neuro-Oncology (RANO) criteria have been proposed in 2010 (18) as a tool to address this issue with a specific version released in 2015 (immunology RANO, iRANO) for patients enrolled in IT protocols (19). They are based on conventional MRI (cMRI), which fails to capture the whole complexity of GBM: The size of enhancing and non-enhancing tissue is not a univocal marker of the dynamics of glioma and immune cell interaction (20). Moreover, with iRANO criteria, TTP can be defined only 6 months after the initiation of treatment, which is a considerable time in light of the survival of short patients (19).

Advanced MRI (aMRI) techniques, including perfusion-weighted imaging (PWI) and diffusion-weighted imaging (DWI), can better describe tumor biology: The former is related to angiogenesis and is usually elevated in malignant tissue, while the latter is an inverse marker of tissue hyper-cellularity (low apparent diffusion coefficient, ADC). As such, they can assist in differentiating PsP from TTP and in predicting response to treatment (20–23). However, imaging evaluation during multimodal treatments, and mostly IT, gains specific adjunctive biases and pitfalls (18) due to the infiltration of immune cells and the contrast enhancement (CE) and vessel permeability increase determined by an immune response.

Treatment response assessment maps (TRAMs) are based on conventional T1-contrast enhanced volumetric sequences performed on ≥1.5-Tesla MR scanners: The technique exploits the principle of delayed contrast imaging as the subtraction of late and early post-contrast scans allows the identification of areas of early contrast-medium clearance (conventionally blue colored, hypothesized to be tumoral, due to neoangiogenic vessels) and areas of contrast-medium accumulation (red, hypothesized as treatment-related). Blue volumes have been histologically validated in patients receiving radiotherapy as a surrogate marker of tumor tissue, while red volumes have been demonstrated to be non-tumoral tissue (24). Hence, TRAMs have been proposed as a simple tool based on cMRI with potentials comparable to aMRI approaches. To date, their application in glioma IT has never been reported in the literature.

The present study aimed to investigate the potential of TRAMs in the definition of GBM response to dendritic cell IT plus SOC, exploring possible association with known biomarkers such as O6-methylguanine-DNA-methyltransferase (MGMT) hypermethylation and to assess their diagnostic value in the distinction of PsP and TTP during IT.

## 2 Materials and methods

### 2.1 Patient selection

We enrolled 16 patients meeting the criteria for the DENDR1 phase II clinical trial (NCT04801147) in patients with newly diagnosed GBM. The Institutional Review Board approved the study (protocol n. 419/2014), and informed consent was obtained for all patients.

Inclusion criteria were as follows: histologically proven IDH1/2-wild type (wt) GBM, age between18 and 70 years, residual contrast-enhancing tumor volume after surgery ≤10 cm^3^ confirmed by postoperative MRI, dexamethasone daily dose ≤4 mg during the 2 days prior to leukapheresis, and Karnofsky Performance Score (KPS) ≥ 70.

Subependymal or multifocal diffusion of the tumor was the exclusion criterion.

### 2.2 Treatment protocol

**Figure 1:**
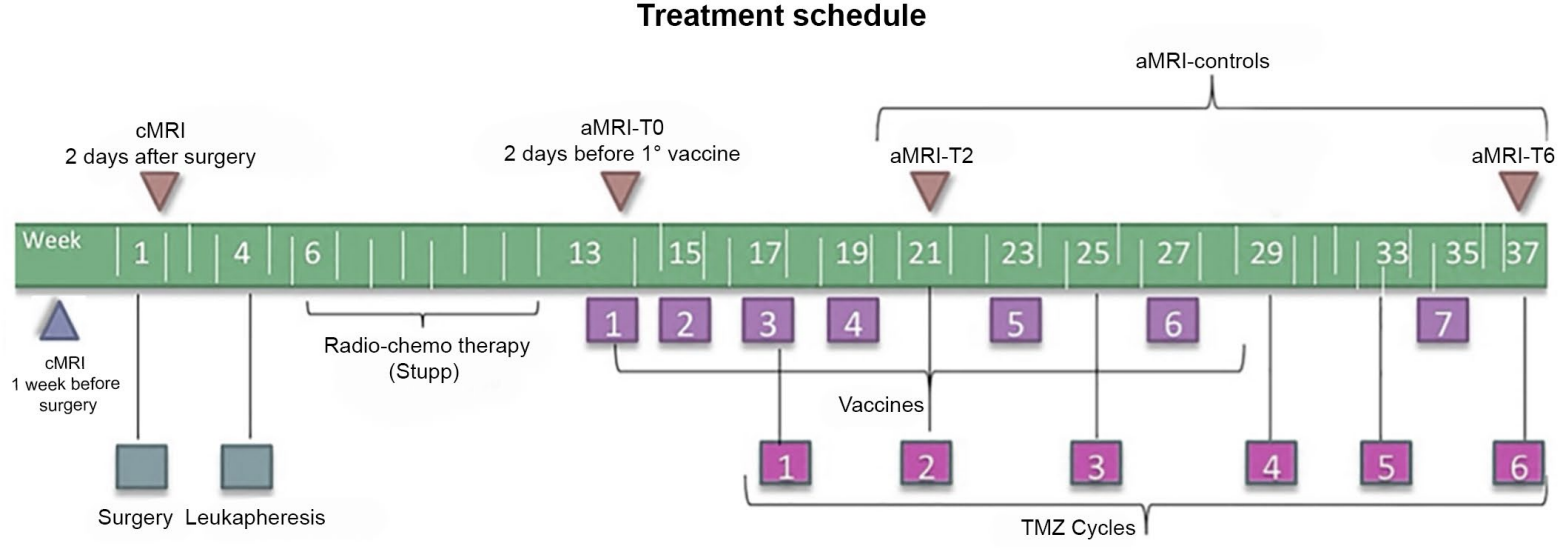
Timeline of the treatment regimen (below) and MRI examinations (above) of patients enrolled in the DENDR1 study protocol. TMZ, temozolomide; aMRI, advanced MRI; and cMRI, conventional MRI.

All patients underwent surgery with subsequent leukapheresis and radiochemotherapy according to the Stupp protocol (2). Subsequently, seven doses of the vaccine were prepared according to the Good Manufacturing Practices (25) and administered as described elsewhere from the same group (12); six doses of TMZ were also administered starting from dose 3 of the vaccine (12). Figure 1 summarizes the treatment schedule.

### 2.3 Imaging follow-up

According to the study protocol (12, 22), patients underwent contrast-enhanced cMRI within 1 week before surgery, within 2 days after surgery, and subsequently cMRI plus aMRI, within 2 days before the first vaccination and then every 2 months or whether clinical worsening occurred. Concomitant clinical monitoring was performed according to the iRANO criteria (19). Time points are displayed in Figure 1.

### 2.4 MRI acquisition

MRI was performed using a Philips 3 T scanner (Achieva TX; Philips Healthcare, Best, the Netherlands) with a 32-channel head coil.

The protocol included the following sequences: (i) 3D fluid attenuation inversion-recovery (FLAIR) (TR/TE = 4,800 ms/333 ms, TI = 1,650 ms, slice thickness = 1 mm, no gap, matrix = 240 × 240, field of view (FOV) = 240 × 240 mm); (ii) axial turbo spin-echo T2-weighted (TR/TE = 2,313 ms/76.5 ms, FA = 90°, slice thickness = 3 mm, matrix = 1,024 × 1,024, FOV = 240 × 240 mm); (iii) single-shot echo-planar DWI (TR/TE = 2,936 ms/62.5 ms, slice thickness = 4 mm, matrix = 288 × 288, FOV = 288 × 288 mm, three orthogonal directions, b = 0–1,000 s/mm2, bi-commissural acquisition) from which ADC maps were automatically reconstructed; (iv) dynamic susceptibility contrast (DSC-MRI) gradient-echo (GRE) (TR/TE = 1,500 ms/40 ms, slice thickness = 5 mm, FA = 75, matrix = 112 × 112, FOV = 224 × 224 mm, Gadovist®,0.1 cc/Kg, 5 mL/s and fixed 3 cc pre-bolus) from which cerebral blood volume (CBV) maps were estimated using nordicICE (NordicNeuroLab AS, Norway, https://www.nordicneurolab.com/help-all/nordicice); (v) 3D-T1 fast-field-echo (FFE) (TR/TE = 9.93 ms/4.5 ms, FA = 8, slice thickness = 1 mm, no gap, matrix = 240 × 240, FOV = 240 × 240 mm) acquired before, 5 min and 75 min after intravenous contrast-medium injection.

### 2.5 MRI post-processing

#### 2.5.1 Volume estimation

The volume of contrast enhancement (V_CE_) of each lesion was manually segmented using MRIcro ver. 1.4.^1^

#### 2.5.2 TRAMs

Estimation of maps requires the acquisition of high-resolution 3D-T1-weighted scans at early (5 min) and late (>1 h) time points after intravenous contrast injection to determine early and late washout to identify tumor tissue from non-specific contrast-enhancing tissue. The subtraction of T1 images acquired 5 min post-contrast from those acquired approximately 75 min after contrast injection yielded the color maps known as TRAMs, which represent the spatial distribution of contrast accumulation/clearance. Blue color represents regions with negative subtraction values, where contrast has been cleared in late enhancement scans, as is the case of abnormal vessels proliferating within the tumor. Red color, conversely, codes for regions with positive subtraction values, where contrast stagnation happens in late scans, as is the case of radiation necrosis or inflammatory tissue.

The timing of post-contrast acquisitions is critical. The choice of the first time point is fundamental because immediately after contrast injection, the gadolinium signal rises fast and it has to be high when the images are acquired in order to be sensitive to tumor regions (conventionally blue-colored). On the other hand, this acquisition time point has to be early enough not to lose sensitivity to treatment effects (conventionally red-colored). The closer to the maximal peak value, the larger is the difference between early and delayed signals.

The choice of the second time point is mainly affected by the time the tumor takes to clear gadolinium from the tissue. Inter- and intra-tumor variability in clearance times exists, but after 1 h, the signal changes slowly; therefore, the second time point can be flexible between 60 and 100 min post-injection (26).

Both early and late post-gadolinium 3D-T1 weighted scans are imported into a dedicated workstation running MATLAB.^2^

Preprocessing of images is essential as described elsewhere, with correction of image intensity values, rigid body, and elastic/local registration (24, 27).

### 2.6 RANO and iRANO criteria volume estimation

The definition of GBM response to therapy is currently based on cMRI by the RANO (18) and iRANO criteria (19) for SOC and IT, respectively. They are both based on two-dimensional measurements of enhancing and non-enhancing tissue changes. We used volumetric measurements, instead of two-dimensional measurements, as more recently suggested (28). We considered as baseline timepoint (T0) the MRI acquired after RT and just before IT, also according to the RANO 2.0 criteria (29). PsP was defined as an increase of enhancing tumor volume by ≥40% during the first 6 months of IT without significant clinical worsening and with stable or regressing lesions at the following MRI without changing therapy (19, 28).

### 2.7 Immune monitoring

Immune monitoring was performed on the peripheral whole blood of each patient before the treatment, after each vaccination, and every 2 months until tumor recurrence as described elsewhere (12).

Briefly, T-cell subsets were monitored by flow cytometry using anti-CD3-VioBlue, anti-CD4-FITC anti-CD8-APC, and anti-CD56-PE monoclonal antibodies (Miltenyi Biotec). The vaccination/baseline (V/B) ratio for NK cell counts was used as a dichotomous parameter. The ratio of the mean of vaccinations (2nd to 7th)/baseline values (V/B ratio) of NK cell absolute count for each naïve GBM patient treated till now with immunotherapy with dendritic cells and the Stupp protocol in our institution (60 cases including the 16 pts. in this study) was calculated, and the median of all the observations was used as the cutoff value to separate patients into the “LOW-NK” (immunological non-responders) or “HIGH-NK” (immunological responders) group (12).

### 2.8 Statistical analysis

The following TRAMs radiological parameters were collected for each patient at different time points until tumor progression: the overall volume of V_CE_, the volume of the red tissue on the map (V_Red_), and the volume of the blue tissue on the map (V_Blue_); we also derived the fraction of red and blue volumes over the V_CE_ (V_Blue_/V_CE_ and V_Red_/V_CE_) and the percentage variation of V_Red_ (ΔV_Red_) and V_Blue_ (ΔV_Blue_) compared to the relative baseline evaluation (calculated as V_Blue_/V_Blue-baseline_ and V_Red_/V_Red-baseline_, respectively).

A non-Gaussian distribution of parameters was assumed. Median was used to describe variables. Changes from the baseline of radiological parameters were assessed using the Wilcoxon–Mann–Whitney test for unpaired samples and the Wilcoxon signed-rank test for paired samples. All *p*-values were two-sided. The same tests were used to determine the significance of differences in radiological parameters between different subgroups of patients (the HIGH- or LOW-NK group; hypermethylated and unmethylated MGMT) or between different phases of the disease (pre-progression and progression).

Progression-free survival (PFS) was calculated from the first surgery until disease progression or death/last follow-up if censored. OS was calculated from surgery to death due to any cause or last follow-up (censored). The Kaplan–Meier analysis was used to estimate PFS and OS. The log-rank test assessed differences in progression or survival in patients with different radiological or clinical parameters.

For radiological parameters, ROC curves were estimated to determine the value of optimal sensitivity and specificity to differentiate responders *vs* non-responders to treatment as other biological subgroups and to distinguish TTP from PsP.

All statistical analyses were performed using SPSS 22.0 for IBM (SPSS Inc., Chicago, IL, USA) software.

## 3 Results

### 3.1 Demographics and immunological parameters

We recruited 16 patients with histologically proven IDH1/2-wt GBM according to the inclusion and exclusion criteria described in the DENDR1 trial (12, 30).

Main demographic and clinical data of patients are summarized in Table 1.

**Table 1:** Main demographic and clinical variables.

Patient	Age	Sex	TMZ cycles	MGMT (Met ≥ 0.1)	Vaccine doses	PFS (months)	OS (months)	2nd surgery	NK V/B ratio	Responder (PFS > 12 months)
**1**	60–69	M	6	M (0.21)	7	16.1	33.6	Y	HIGH	Y
**2**	50–59	M	6	U (0.04)	7	16.4	38.4	Y	HIGH	Y
**3**	50–59	M	6	M (2.39)	7	12.0	23.3	Y	HIGH	N
**4** ^**†**^	50–59	F	6	U (0.00)	7	20.1	28.0	N	HIGH	Y
**5** ^**†**^	40–49	M	6	M (1.51)	7	14.0	20.2	N	LOW	Y
**6** ^**†**^	40–49	F	6	M (0.28)	7	15.1	22.6	Y	HIGH	Y
**7** ^°^	50–59	M	6	M (1.02)	7	7.1	29.7	Y	LOW	N
**8** ^°^	60–69	M	1	U (0.01)	4	3.1	8.5	N	LOW	N
9*	50-59	M	6	U (0.09)	7	37.7	37.7	N	HIGH	Y
**10** ^°^	50–59	M	2	M (1.86)	4	6.0	11.5	N	LOW	N
**11** ^§°^	60–69	F	3	U (0.00)	6	5.3	20.4	Y	HIGH	N
**12**	50–59	M	6	M (0.76)	7	14.2	24.9	Y	HIGH	Y
**13**	40–49	M	6	U (0.01)	7	16.7	23.8	N	HIGH	Y
**14** ^°^	50–59	M	4	U (0.00)	6	5.3	15.7	N	LOW	N
**15**	60–69	M	6	M (0.95)	7	16.7	28.8	N	HIGH	Y
**16**	60–69	M	6	U (0.01)	7	10.6	23.8	N	LOW	N

^*^Patient exitus before progression because of heart failure; ^§^Patient with pathological evidence of mixed treatment-related effects and tumor cells; ^†^Patients with evidence of pseudoprogression (PsP) before true tumor progression (TTP). °Patients 7, 8, 10, 11, and 14 were not included in month 6 aMRI. MGMT, O6-methylguanine-DNA-methyltransferase; PFS, free progression-free survival; OS, overall survival; NK, natural killer; V/B, vaccination/baseline.

There were 13 men and 3 women; the median age was 58 years.

All patients were followed up until progression or death (median follow-up of 23.7 months). Only 1 patient died before progression (due to heart failure), 15 patients experienced progression, and 7 of them underwent second surgery with histopathological confirmation of recurrent tumor in 6 cases while evidence of mixed sample of tumor cells plus treatment-related effects in 1 case. The median PFS was 14 months. Three patients experienced PsP before evidence of TTP. Nine patients were free from progression at 12 months: Here, for brevity and clarity, we defined them as responders. All patients were dead at the data analysis time, and the median OS was 24 months. PFS and OS in responder patients were significantly longer than in non-responder cases (PFS 6 months vs. 16.4, *p* = 0.0001; OS 20.4 vs. 28.8, *p* = 0.013).

In total, 10 patients were defined as HIGH-NK and 6 as LOW-NK according to the immunological monitoring of peripheral blood. In total, five of seven patients with an early TTP (within 7 months of follow-up) were in the LOW-NK group. PFS and OS in the HIGH-NK patients were significantly longer than in the LOW-NK group (PFS 6 vs. 16.4, *p* = 0.0001; OS 20.2 vs. 28.8, *p* = 0.004).

Hypermethylation of the MGMT promoter, evaluated by methylation-specific PCR (polymerase chain reaction) (30), was detected in 8 of the 16 patients.

### 3.2 TRAMs

#### 3.2.1 Analyses in all patients

The main TRAM parameters are summarized in Table 2.

**Table 2:** Tumor volumes (cm^3^) of all patients at baseline (day 0), month 2, and month 6.

	Baseline (*n* = 16)	2 months (*n* = 16)	6 months (*n* = 11)
V_CE_	7.35 (1.4–51.7)	7.05 (0.1–78.8)	**2.8 (0–9.5)** ^**§†**^
V_Blue_	2.95 (0.3–27.8)	3.45 (0–46.3)	**1.2 (0–8.3)**
V_Red_	1.6 (0.4–13.9)	2.25 (0–24.6)	**1.3 (0–2.7)**
V_Blue_/V_CE_	0.57 (0.19–0.87)	**0.52 (0.06–0.68)** ^**§**^	0.43 (0.22–0.69)
V_Red_/V_CE_	0.28 (0.09–0.65)	0.32 (0.06–0.75)	0.32 (0.07–0.6)
ΔV_Blue_	1	0.75 (0.028–4.62)	**0.23 (0.034–0.7)** ^**§†**^
ΔV_Red_	1	0.7 (0.031–6.26)	0.17 (0.006–2.2)

^§^*p* < 0.05 compared to baseline; ^†^*p* < 0.05 compared to 2 months. V_CE_, V_Blue_, and V_Red_ are, respectively, the volumes of contrast enhancement (CE) and the blue and the red area volumes of the tumor. The bold terms represent values with *p*<0.05.

A statistically significant decline in the median of the overall volume V_CE_ was observed by comparing values detected 6 months after IT to those at baseline and month 2 (*p* = 0.005). In addition, the median of V_Blue_ (*p* = 0.03 and *p* = 0.013, respectively) and ΔV_Blue_ (*p* = 0.003 and *p* = 0.021) were significantly reduced at month 6 compared to both previous time points. The fraction of blue volume (V_Blue_/V_CE_) was significantly reduced at month 2 compared to baseline (*p* = 0.008).

The median red volumes did not change significantly at month 2, and later on, V_Red_ declined at month 6 compared to month 2 (*p* = 0.033).

#### 3.2.2 Analyses in TTP and PsP

In the 15 patients who experienced TTP and in the three who showed PsP during the follow-up, we compared TRAM parameters observed, respectively, at TTP or PsP to those detected in the immediately previous MRI examination (Supplementary Table S1). Due to a small number of patients, a formal statistical analysis of TRAM parameters in PsP was not performed.

The median V_CE_ increased in both TTP (*p* = 0.009) and PsP.

In TTP, median V_Blue_ and, slightly, median V_Red_ increased (*p* = 0.007 and *p* = 0.05, respectively); however, after normalization to baseline values, only ΔV_Blu_ showed a significant increase (*p* = 0.013).

In PsP, median V_Blue_ also increased, but the blue volume fraction (V_Blue_/V_CE_) decreased.

Using ROC curves, a threshold of −0.066 in V_Blue/_V_CE_ variation between values obtained in the MRI performed immediately before PsP/TTP and at TTP/PsP allowed to discriminate TTP from PsP with a sensitivity of 71.4% and specificity of 100% (area under the curve, AUC: 0.875; *p* = 0.001) (Supplementary Figure S1). Accordingly, if a decrease ≥0.06 in V_blu_/V_CE_ was observed, the patient is predicted to have a pseudoprogression.

**Figure 2:**
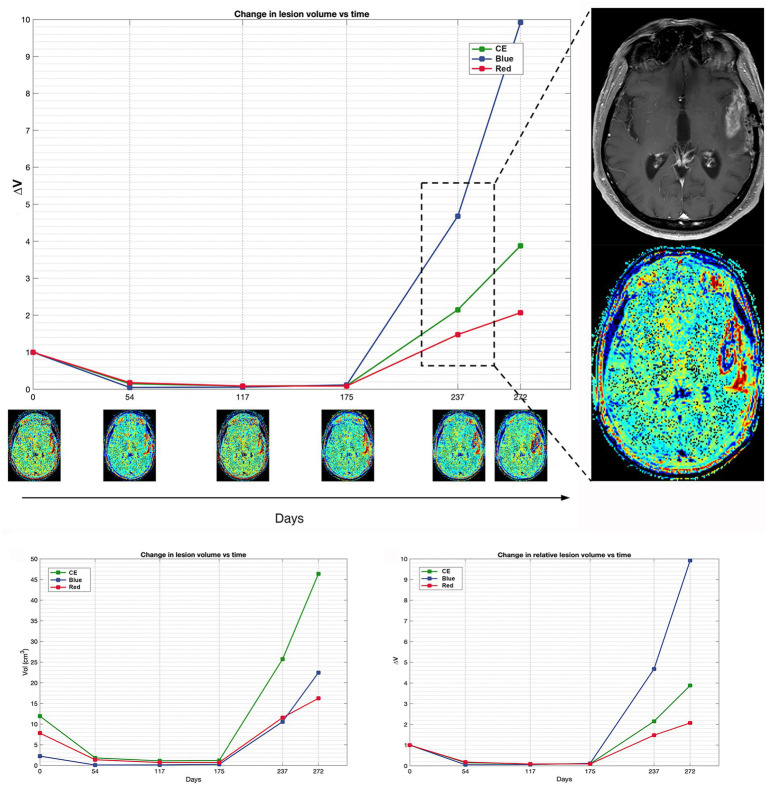
Patient 16 exhibits a classic scenario of TTP at day 237, demonstrating a notable increase in blue volume compared to the pre-progression MRI at day 175. The magnification on the right displays the treatment response assessment maps (TRAMs) of day 237 alongside the corresponding post-contrast T1 image, highlighting a significant presence of blue color within the contrast-enhancing (CE) region. Additionally, a comparison of absolute and relative volumes between patients with PsP and classic TTP underscores how relative volumes (ΔV_Red_ and ΔV_Blue_, respectively) better illustrate the increase in the red component observed in PsP and the escalation of the blue component evident in TTP.

**Figure 3:**
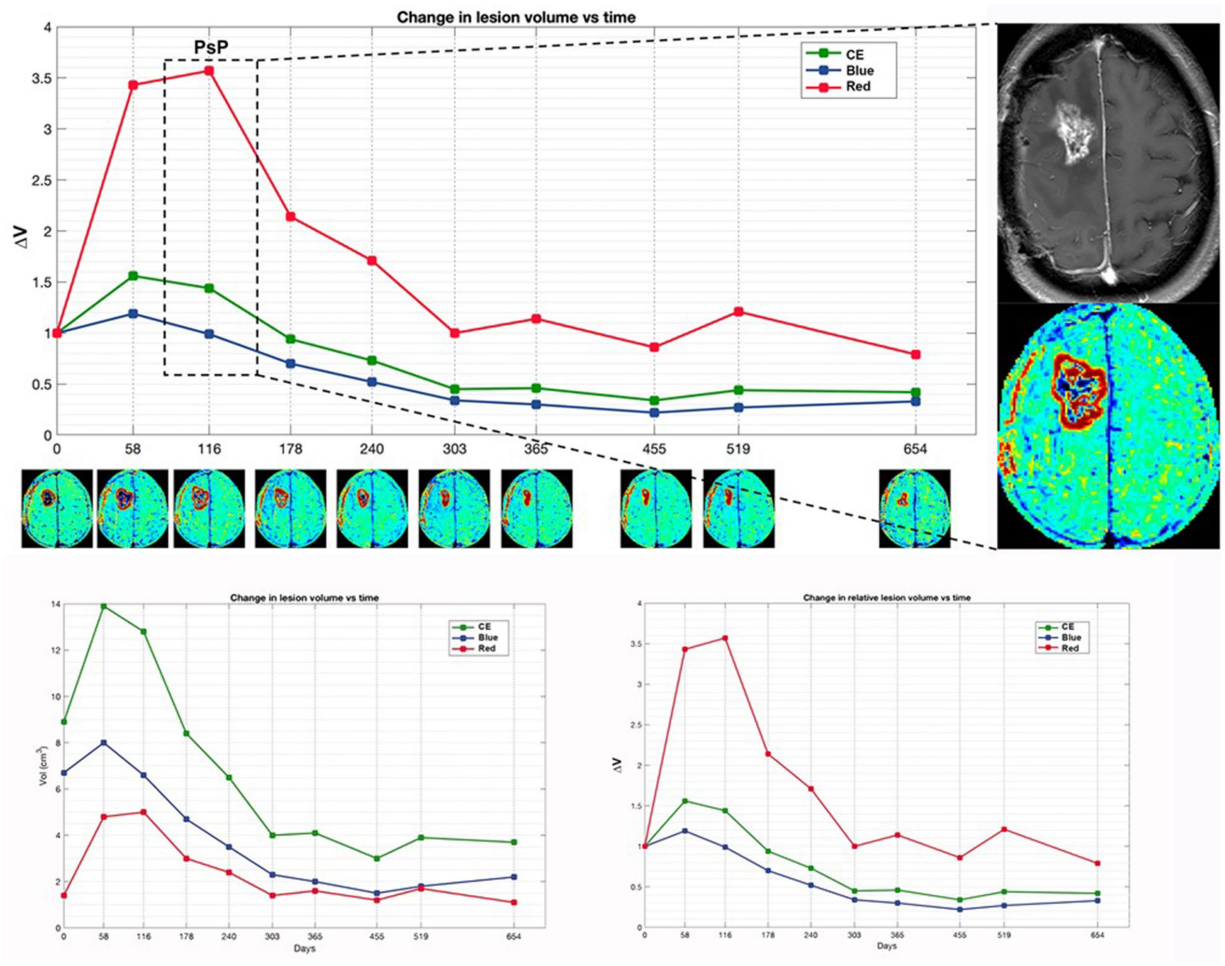
Patient 4 with initial suspected PsP exhibits a preponderant increase in red volume in early time points, coinciding with the suspicion of PsP. Subsequently, TTP manifests at a later time point, marked by the appearance of a new distant lesion in the contralateral thalamus (not shown). The magnification on the right displays the treatment response assessment maps (TRAM) during the PsP phase, alongside the corresponding post-contrast T1 image. Additionally, a comparison of absolute and relative volumes between patients with PsP and classic TTP, as illustrated in Figure 4, underscores how relative volumes (ΔV_Red_ and ΔV_Blue_, respectively) better demonstrate the increase in the red component observed in PsP and the escalation of the blue component evident in TTP.

Figures 2, 3 display differences in TTP and PsP in two patients.

#### 3.2.3 Analyses in responder and non-responder patients

No statistically significant differences were detected between any median of the baseline TRAM parameters detected in responder *vs* non-responder patients (Supplementary Table S2).

Among non-responder patients, after 2 months of treatment, the median fraction of V_Red_ (i.e., V_Red_/V_CE_) was increased from baseline (*p* = 0.018). Noteworthy, at month 6 only 2 non-responder patients were progression-free and still on follow-up (Supplementary Table S3).

Among responder patients, at month 6, there was a significant reduction compared to both baseline and month 2 in median V_CE_ (*p* = 0.028 and *p* = 0.012), V_Blue_ (*p* = 0.008 and *p* = 0.017), and ΔV_Blue_ (p = 0.008 and p = 0.028, respectively) (Supplementary Table S3).

At month 2, using ROC curves the threshold for discriminating responder *vs* non-responder patients for V_Red_/V_CE_ variation was ≤0.001 with a sensitivity of 66.7% and specificity of 100% (AUC: 0.754; *p* = 0.059). Accordingly, if a reduction ≤0.001 in V_Red_/V_CE_ was observed, the patient is predicted to be a non-responder case (Supplementary Figure S2).

At month 6, using ROC curves the threshold for discriminating responder *vs* non-responder patients for V_blue_/V_CE_ variation was ≤0.035 with 77.8% sensitivity and 100% specificity (AUC 0.88 *p* = 0.04). Accordingly, if a reduction ≤0.035 in V_Blue_/V_CE_ was observed, the patient is predicted to be a non-responder case (Supplementary Figure S3). Using this threshold in dividing patients, we observed a statistically significant difference in OS (29.9 vs. 23.8, *p* = 0.009), suggesting the benefit of delayed-contrast MR imaging in predicting treatment response.

#### 3.2.4 Analyses in the HIGH-NK and low-NK patients

No statistically significant differences were detected between any median of the baseline TRAM parameters of the HIGH-NK and LOW-NK patients.

After 2 months of treatment, the median ΔV_Red_ was higher in the HIGH-NK (n = 9) patients than the LOW-NK (n = 7) patients (*p* = 0.031).

Among the LOW-NK patients, no significant changes in TRAM parameters were detected after 2 and 6 months of treatment, apart from a mild reduction of median V_Blue_/V_CE_at the second month (*p* = 0.043). Noteworthy, at month 6, only 2 LOW-NK patients had not yet undergone progression.

Among the HIGH-NK patients, at month 6, there was a significant reduction compared to both baseline and month 2 in median V_CE_ (*p* = 0.015 and *p* = 0.012), V_Blue_ (*p* = 0.008 and *p* = 0.017), and ΔV_Blue_ (*p* = 0.008 and *p* = 0.021, respectively). Analyses in HIGH-NK and LOW-NK patients are displayed in Table 3.

**Table 3:** Tumor volumes (cm^3^) in the HIGH-NK and the LOW-NK patients at baseline, month 2, and month 6.

	Baseline		2 months		6 months	
	HIGH (*n* = 9)	LOW (*n* = 7)	HIGH (*n* = 9)	LOW (*n* = 7)	HIGH (*n* = 9)	LOW (*n* = 2)
V_CE_	7 (1.4–23.7)	12 (0.4–51.7)	6.5 (0.1–30.7)	7.6 (1.1–78.8)	**2.8 (0.6–15.4)** ^†*^	6.65 (1.2–12.1)
V_Blue_	3.5 (0.6–15.4)	2.4 (0.3–27.8)	3 (0–16.7)	4.2 (0.1–46.3)	**1.2 (0–4.7)** ^†*^	4.3 (0.3–8.3)
V_Red_	1.5 (0.5–11.2)	4.3 (0.1–13.9)	2 (0–7.5)	2.5 (0.3–24.6)	1.3 (0–3)	1.4 (0.7–2.1)
V_Blue_/V_CE_	0.43 (0.28–0.87)	0.61(0.19–0.76)	0.43 (0.21–0.6)	0.56 (0.059–0.68)	0.43 (0.32–0.75)	0.46 (0.22–0.69)
V_Red_/V_CE_	0.37 (0.09–3)	0.27 (0.13–0.65)	0.26 (0.065–0.63)	0.32 (0.26–0.75)	0.32 (0.073–0.51)	0.38 (0.17–0.76)
ΔV_Blue_	1	1	**0.54 (0.028–1.19)** ^**†**^	1.52 (0.048–4.62)	**0.23 (0.034–0.7)** ^†*^	0.41 (0.12–0.7)
ΔV_Red_	1	1	**1.52 (0.18–6.26)** ^**§**^	0.67 (0.031–3.6)	0.29 (0.09–0.49)	0.17 (0.006–2.2)

^§^*p* < 0.05 compared to the LOW-NK at the same time point. ^†^*p* < 0.05 compared to baseline (same group). ^*^p < 0.05 compared to month 2 (same group). The bold terms represent values with *p*<0.05.

Using ROC curves, the threshold for discriminating LOW *vs* HIGH-NK patients for V_Red_/V_CE_ variation at 6 months was ≥ − 52 with a sensitivity of 87.5% and specificity of 100% (AUC 0.875 *p* = 0.003) Accordingly, if a reduction >52 in V_Red_/V_CE_ was observed, the patient is predicted to be a LOW-NK case (Supplementary Figure S4).

No statistically significant differences were detected between any median TRAM parameters at baseline and during treatment between patients with hypermethylated or unmethylated MGMT.

#### 3.2.5 Illustrative case of a mixed scenario

**Figure 4:**
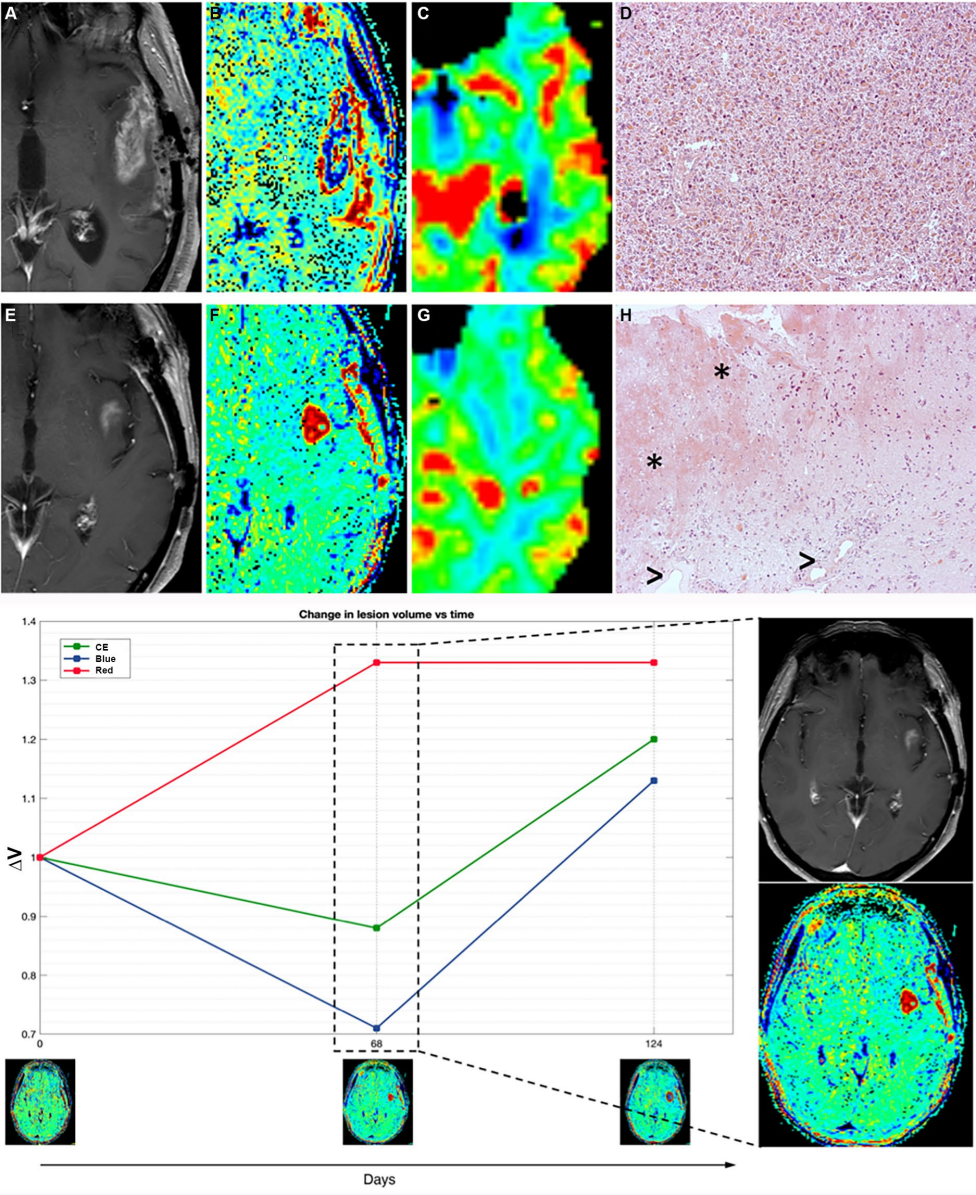
Patient 11 **(E–H)** presented with suspected recurrence due to the emergence of a newly enhancing lesion in the left insula at day 68, leading to subsequent surgery revealing histopathological evidence of treatment-related effects and glioma cells. Retrospectively, TRAMs at that time point primarily indicated an increase in red volume, suggestive of non-tumoral tissue, as observed both in the plot (left) and in the visual maps (right). In comparison with a case of TTP (true tumor progression) (**A–D**, patient 16), where an enhancing lesion with high cerebral blood volume (CBV) and prevalence of V_Blue_ on TRAMs corresponded histopathologically to glioblastoma multiforme (GBM) recurrence, Patient 11 **(E–H)** exhibited an enhancing lesion with moderately high CBV and a prevalence of V_Red_ on TRAMs. Histopathological examination, in patient 16 with TTP **(D)**, showed densely cellular neoplasia composed of elements with marked polymorphism, frequent mitoses, and the presence of vascular proliferation, consistent with glioblastoma multiforme (GBM) recurrence, while in patient 11 (mix scenario, **H**) revealed nervous tissue with treatment-related alterations, including coagulative necrosis and gliosis (*), along with vessels exhibiting thick hyaline walls and perivascular inflammatory infiltrates (>), and atypical glial elements indicative of infiltration by high-grade glioma.

Patient 11 in our cohort precociously interrupted IT due to the appearance of a new enhancing lesion in the left insula showing moderate hyperperfusion, suspected for recurrence (Figure 4).

TRAMs at the time of suspected progression mainly showed an increase in volumes of red (Figure 4).

She underwent a new surgery with histopathological evidence of treatment-related effects and rare glioma cells (Figure 4). In the patients with such histological findings (as patient 11), the alterations of the endothelium, vascular walls, and blood–brain barrier enable perivascular inflammation and support the imaging features.

## 4 Discussion

GBM has a dismal prognosis in most cases, besides multimodal standardized and experimental treatments.

IT is a quite novel treatment modality for GBM, known to be an immune-suppressive tumor, to induce inflammatory response within the tumor environment. To date, SOC for the GBM treatment approach relies on radiotherapy and TMZ chemotherapy according to the Stupp protocol; thus, IT can be added to SOC but cannot replace it.

Confident and early identification of TTP is vital to avoid the continuation of non-effective therapies and possibly to switch to an alternative treatment regimen. Standard accepted response assessment criteria (iRANO), based on cMRI, are not able to univocally discriminate between TTP and PsP, which is a non-tumoral radiological expression of treatment-related tissue inflammatory alteration. Moreover, there is no validated MRI surrogate marker of immunological response, even if changes in CBV, k_trans_, and ADC have been proposed as possible ones (20, 22).

The patients who underwent a second surgery and had a clear TTP at the histological examination showed the presence of neoplastic tissue with high cell density, striking pleomorphism, and vascular proliferation, with only rare inflammatory infiltrates.

TRAMs are based on cMRI early and delayed T1-contrast-enhanced volumetric sequences and can be easily performed on ≥1.5-Tesla scanners: The technique has been histologically validated in patients receiving radiotherapy, with “blue” volume as a surrogate marker of tumor tissue, while “red” volumes have been demonstrated to be non-tumoral tissue (24). TRAMs have never been reported in the literature as a possible tool to address these issues in patients undergoing IT. In our pilot study, we applied the TRAMs technique in a homogeneous cohort of 16 patients with naïve GBM IDHwt treated with surgery followed by dendritic cell-based IT added to SOC.

In TTP median V_Bleu_ and, slightly, median V_Red_ increased; however, after normalization to baseline values, only ΔV_Blue_ increase was significant. In PsP, median V_Blue_ also increased, but the fraction of blue volume over the V_CE_ (V_Blue_/V_CE_) decreased, and using ROC curves, a threshold of −0.06 in V_Blue_/V_CE_ variation was able to discriminate TTP and PsP.

An increase in V_Blue_ would be expected in cases of TTP as the blue region, which is hypothesized to correspond to tumor tissue, is the one that should rise most significantly upon progression. However, our data suggest that not the raw data, but the entity of the variation of the fraction of V_Blue_ over the V_CE_ should be considered.

Biases and pitfalls in radiological assessment of response during IT are peculiar and add challenges to multimodal treatment mix scenarios and to GBM which is a non-homogeneous tumor on its own (17).

Other advanced MRI techniques have already been studied as a possible early marker of progression in the setting of GBM and IT: On PWI, elevated CBV values within a region of contrast enhancement have been shown to support a diagnosis of TTP (21, 31), while reduced CBV in the context of an enhancing lesion in GBM has been proposed as a possible marker of PsP (32, 33). However, evaluation of CBV and other DSC-MRI parameters is limited by the location of the lesion as cortical lesions suffer from the physiological high perfusion of the cortex that might mask tumor perfusion; moreover, PWI has a quite low spatial resolution and might miss small lesions. On the other hand, dynamic contrast enhancement (DCE-MRI) K_trans_ is not affected by tumor location, because it is based on T1 sequences with scarce susceptibility to artifacts, particularly near the skull base or at brain–bone–air interfaces, but is related to tumor vessel permeability, which may also be impaired by loosening of endothelial tight junctions due to inflammation in IT. Finally, no clear univocal cutoff for aMRI values has been defined to differentiate TTP from PsP, which often overlap.

The TRAMs are simple to acquire as they only need high-quality 3D-T1 imaging, which has high resolution and does not have artifacts near the cortex nor suffer from susceptibility phenomena. Moreover, they are potentially easier to interpret as late enhancement is either present or absent. The main issues of the technique are the determination of the threshold to discriminate red *vs* blue tissue to obtain adequate maps; the validation of the “red tissue” (hypothesized to be present in PsP or mixed scenarios) on surgical specimens because second surgery in GBM is usually performed only in selected cases and when TTP is strongly suspected. All of the six patients in our study who underwent second surgery due to suspect TTP and had a prevalence of V_Blue_ on TRAMs gained histological diagnosis of recurrence. Nevertheless, the presence of residual both blue and red volumes in many patients in our cohort indicates that tumor cells and inflammatory infiltrates probably coexist in the same patient; as highlighted by the case, we anecdotally displayed with histological evidence of both treatment-related effects and a minority of tumor cells (patient 11, Figure 4) and prevalent V_Red_ on TRAMs before second surgery.

The TRAMs could also provide additional information regarding cases responsive to IT. Only in responder patients, at month 6, we observed a significant decrease in median V_Blue_ and ΔV_Blue_ compared to both baseline and month 2 values. Furthermore, at the same time point, a threshold ≤0.035 for V_Blue_/V_CE_ variation was able to discriminate responder *vs* non-responder cases. The value of this finding was also confirmed using log rank, showing statistically significant differences in OS. Detecting the radiological and immunological characteristics of responder cases will provide valuable information for guiding the optimization of future treatments.

In a previous study from our group, dendritic cell vaccination induced a significant, persistent activation of NK cells associated with prolonged survival (12). We therefore stratified our patients in the HIGH-NK (immunological responders) and LOW-NK (immunological non-responders) groups as previously described (12). No difference in CE or TRAMs at baseline was detected between LOW-NK and HIGH-NK patients. All patients with early progressive disease (<7 months) in our cohort belonged to the LOW-NK category and had a shorter OS, too. HIGH-NK patients had a significant reduction in V_CE_, V_Blue_, and ΔV_Blue_ at month 6 compared to both previous time points, and at month 2 compared to baseline interpretable as a reduction in tumor volume and, therefore, as an indirect sign of tumor response to IT in HIGH-NK patients as opposed to LOW-NK patients, who mostly undergo early progression.

Moreover, only HIGH-NK patients had a trend to increase in red volumes at month 2 compared to baseline: We hypothesize that it could represent an initial increase in non-tumoral enhancement due to an inflammatory response with immune cell infiltrates. The HIGH-NK patients do indeed have a better response to therapy than the LOW-NK patients, as witnessed by their longer PFS and OS. In previous study on a larger cohort of patients including the ones of the present study, we reported that after the 4th dose of vaccine (i.e., 2 months from the beginning of IT) a reduction in minimum ADC values was visible only in the HIGH-NK patients and not in the LOW-NK patients. We attributed this phenomenon to an increased cellularity in the affected tissue due to an immune infiltrate. We therefore now hypothesize that the apparent increase in red volumes at month 2 in TRAMs and the concomitant reduction in minimum rADC might be different features of the same cellular infiltrate that is at the basis of immune response in the HIGH-NK patients as opposed to the LOW-NK patients. The ROC curve analysis confirms that changes in the V_Red_/V_CE_ correlate with an elevated peripheral blood NK cell count, as a possible marker of immune response.

Our enrollment of patients meeting the criteria for the DENDR1 trial, i.e., small residual enhancing volume, high clinical performance, and absence of dissemination and of multifocality, may give reason to the mOS observed in the present study, which was relatively long even in non-responders compared to historical controls, at 20.4 months.

Identification of early imaging markers of tumor response is at least as important as the early discrimination between TTP and PsP: It could help clinicians to better tailor therapies and could have a potential role in the assessment of tumor response in clinical trials. Most of the MRI studies have focused on the distinction between TTP and PsP rather than on the identification of markers of tumor response. Reduced ADC values, which have been proposed as a marker of immune cell infiltration and tumor response, could also be detected in case of progressive disease due to tumor cellularity (21); concomitant evaluation of ADC and CBV can assist in defining the correct scenario. Our pilot study demonstrates that TRAMs might be a potential alternative or an additional tool in the distinction between TTP and PsP, and they might provide early markers of tumor response.

## 5 Conclusion

Our considerations are derived from a pilot study with patients on experimental treatments and are therefore intrinsically limited by the low sample size, which includes the dropout of most clinical and immunological non-responder patients at the 6-month aMRI time point. The statistical power of our analyses is therefore limited. We chose not to include DWI and DSC values in the present pilot paper focused on TRAMs because the number of patients is too small to perform further subgroup of patients. Thus, it is important to further study TRAMs in larger cohorts of patients on similar treatments, ideally with the concomitant use of additional aMRI in order to compare the diagnostic and prognostic values of different imaging techniques, and with histological validation when feasible. In fact, we previously published a study focused on DWI and DSC in GBM immunotherapy (22) and we are collecting more patients with both DWI, DSC, and TRAMs. The present study is preliminary to the RF study “Radiomics, circulating biomarkers and transcriptomics to dissect immune responses to radiotherapy and immunotherapy of glioblastoma” approved by the Italian Ministry of Health (RF-2019-12371008).

## Data availability statement

The raw data supporting the conclusions of this article will be made available by the authors, without undue reservation.

## Ethics statement

The studies involving humans were approved by Review Board of Istituto Neurologico “Carlo Besta” IRCCS. The studies were conducted in accordance with the local legislation and institutional requirements. The participants provided their written informed consent to participate in this study.

## Author contributions

VC: Conceptualization, Formal analysis, Investigation, Methodology, Project administration, Supervision, Validation, Writing – original draft, Writing – review & editing. FS: Data curation, Investigation, Visualization, Writing – original draft, Writing – review & editing. YM: Formal analysis, Methodology, Software, Validation, Writing – review & editing. DL: Formal analysis, Methodology, Software, Validation, Writing – review & editing. SP: Formal analysis, Funding acquisition, Methodology, Resources, Validation, Writing – review & editing. FM: Data curation, Writing – review & editing. MB: Funding acquisition, Resources, Writing – review & editing. EA: Investigation, Writing – review & editing. BP: Formal analysis, Validation, Writing – review & editing. LM: Formal analysis, Writing – review & editing. CR: Data curation, Investigation, Writing – review & editing. EB: Data curation, Writing – review & editing. VP: Methodology, Writing – review & editing. ME: Writing – review & editing, Formal analysis, Funding acquisition, Investigation, Methodology, Project administration, Resources, Supervision, Writing – original draft, Writing – review & editing. DA: Conceptualization, Methodology, Software, Visualization, Writing – original draft, Writing – review & editing.

## Footnotes

^1^ https://people.cas.sc.edu/rorden/mricro/mricro.html#Installation

^2^ https://www.mathworks.com
