# Supplementary material for: Response assessment of GBM during immunotherapy by delayed contrast treatment response assessment maps
Source: Front Neurol. 2024 Apr 8;15:1374737. doi: 10.3389/fneur.2024.1374737 (PMC11033465; doi:10.3389/fneur.2024.1374737)
Supplement: Supplementary file 1 [file Table_1.DOCX]

Supplementary Material

Response assessment of GBM during immunotherapy by delayed contrast TRAMs (treatment response assessment maps)

Valeria Cuccarini^1^, Filippo Savoldi^1^, Yael Mardor^2,3^, David Last^2^, Serena Pellegatta^4^, Federica Mazzi^1*^, Maria Grazia Bruzzone^1^, Elena Anghileri^4^, Bianca Pollo^5^, Luisa Maddaloni^4^, Camilla Russo^6^, Elisa Bocchi^1^, Valentina Pinzi^7^, Marica Eoli^4*^, Domenico Aquino^1^

*** Correspondence:**  Marica Eoli: marica.eoli@istituto-besta.it

Federica Mazzi: [federica.mazzi@istituto-besta.it](mailto:federica.mazzi@istituto-besta.it)

# Table 1S. Median TRAM parameters measured at TTP (True Tumor Progression) and PsP (Pseudoprogression) time and in the immediately previous MRI exam

|  | **Pre-TTP (15)** | **TTP (15)** |  | **Pre- PsP (3)** | **PsP (3)** |
| --- | --- | --- | --- | --- | --- |
| **V CE (cm^3^)** | 2.65 (0.4-51.7) | 4.45 (1.1-78.8) | 0.009 * | 7.7 (1.9-20.5) | 14.3 (4.3-30.7) |
| **V Blue (cm^3^)** | 1.1 (0.2-27.8) | 2.25 (0.2-46.3) | 0.007 * | 11.7 (6.7-11.4) | 9.3 (8-16.7) |
| **V Red (cm^3^)** | 0.95 (0.1-13.9) | 1.65 (0-24.6) | 0.05 * | 3.1 (1.4 - 4.3) | 3.1 (2-4.8) |
| **Vol.Blue/Vol.CE** | 0.53 (0.23-0.77) | 0.50 (0.17-0.72) | 0.43 | 0.87 (0.65-6) | 0.56 (0.54-2.7) |
| **Vol.Red/Vol.CE** | 0.32 (0.13-0.60) | 0.33 (0-0.46) | 0.28 | 0.18 (0.15-2.2) | 0.34 (0.06-0.91) |
| **Vol.Blue/Vol.Blue _(day0)_** | 0.37 (0.08-1) | 0.60 (0.15-4.68) | 0.013 * | 1 | 1.08 (0.79-1.19) |
| **Vol.Red / Vol.Red _(day0)_** | 0.73(0.04-2.2) | 1.19 (0-6.26) | 0.023 * | 1 | 0.72 (0.64-3.42) |

V_CE_, V_Blue_, V_Red_ are respectively the volumes of contrast enhancement (CE), the blue and the red area of the tumor

* p<0.05

# Table 2S. Median TRAM parameters measured at different time points (Baseline – Two months – Six Months) in responder vs non-responder patients

|  | **Baseline** | |  | **Two-months** | |  | **Six-months** | |  |
| --- | --- | --- | --- | --- | --- | --- | --- | --- | --- |
|  | **Responders (9)** | **Non responders (7)** | **p** | **Respoders (9)** | **Non responders (7)** | **p** | **Responders (9)** | **Non responders (2)** | **p** |
| **V CE (cm^3^)** | 7.7 (1.4-23.7) | 4 (0.4-30.7) | 0.68 | 10 (0.1-30.7) | 3.5 (1.1-78.8) | 0.91 | 4.6 (0-12.1) | 0.8 (0.4-1.2) | 0.43 |
| **V Blue (cm^3^)** | 6.7 (0.6-15.4) | 2.3 (0.3-27.8) | 0.75 | 3.9 (0-16.7) | 1.7 (0.3-27.8) | 0.83 | 1.8 (0-4.7) | 0.3 (0.3) | 0.58 |
| **V Red (cm^3^)** | 1.7 (0.5 11.2) | 1.2 (0.1-13.9) | 0.75 | 2.7 (0-7.5) | 1.6 (0.3-24.6) | 1 | 2.1 (0-3) | 0.48 (0.22-0.75) | 0.32 |
| **Vol.Blue/Vol.CE** | 0.5 (0.28-0.87) | 0.6 (0.19-0.76) | 0.75 | 0.51 (0.06-0.6) | 0.53 (0.059-0.61) | 0.47 | 0.43 (0.32-0.68) | 0.46 (0.22-0.69) | 1 |
| **Vol.Red/Vol.CE** | 0.35 (0.09-0.52) | 0.27 (0.13-0.65) | 0.75 | 0.26 (0.065-0.63) | 0.33 (0.25-0.75) | 0.3 | 0.31 (0.073-0.51) | 0.42 (0.25-0.6) | 0.58 |
| **Vol.Blue/Vol.Blue_(day0)_** | 1 | 1 | 0.17 | 0.56 (0.028-1.19) | 1.52 (0.048-4.62) | 0.14 | 0.25 (0.034-0.7) | 0.17 (0.12-0.23) | 0.58 |
| **Vol.Red/Vol.Red_(day0)_** | 1 | 1 | 0.17 | 1.52 (0.17-6.26) | 1.64 (018-6.26) | 0.29 | 0.48 (0.006-2.2) | 0.12 (0.09-0.16) | 0.58 |

V_CE_, V_Blue_, V_Red_ are respectively the volumes of contrast enhancement (CE), the blue and the red area of the tumor

* p<0.05

# Table 3S. Median TRAM parameters measured in responder and in non-responder patients comparing different time points (Baseline – Two months – Six Months) in each subgroup

|  | **Responders** | | | | | | **Non responders** | | |
| --- | --- | --- | --- | --- | --- | --- | --- | --- | --- |
| **Responders** | **Baseline** | **Two-months** | **p** | **Six-months** | **Six months vs baseline** | **Six vs Two months** | **Baseline** | **Two-months** | **p** |
| **V CE (cm^3^)** | 7.7 (1.4-23.7) | 10 (0.1-30.7) | 0.67 | 4.6 (0-12.1) | 0.028 * | 0.012 * | 4 (0.4 -30.7) | 3.5 (1.1-78.8) | 0.49 |
| **V Blue (cm^3^)** | 6.7 (0.6-15.4) | 3.9 (0-16.7) | 0.21 | 1.8 (0-4.7) | 0.008 * | 0.017 * | 2.3 (0.3 -27.8) | 1.7 (0.3-27.8) | 1 |
| **V Red (cm^3^)** | 1.7 (0.5-11.2) | 2.7 (0-7.5) | 0.44 | 2.1 (0-3) | 0.20 | 0.06 | 1.2 (0.1 -13.9) | 1.6 (0.3-24.6) | 0.27 |
| **Vol.Blue/Vol.CE** | 0.5 (0.28-0.87) | 0.51 (0.06-0.6) | 0.38 | 0.43 (0.32-0.68) | 0.08 | 0.59 | 0.6 (0.19-0.76) | 0.53 (0.059-0.61) | 0.063 |
| **Vol.Red/Vol.CE** | 0.35 (0.09-0.52) | 0.26 (0.065-0.63) | 0.67 | 0.31 (0.073-0.51) | 0.95 | 0.95 | 0.27 (0.13-0.65) | 0.33 (0.25-0.75) | 0.018 * |
| **Vol.Blue/Vol.Blue_(day0)_** | 1 | 0.56 (0.028-1.19) | 0.051 | 0.25 (0.034-0.7) | 0.008 * | 0.028 * | 1 | 1.52 (0.048-4.62) | 0.31 |
| **Vol.Red/Vol.Red_(day0)_** | 1 | 1.52 (0.17-6.26) | 0.51 | 0.48 (0.006-2.2) | 0.67 | 0.13 | 1 | 1.64 (018 -6.26) | 0.23 |

V_CE_, V_Blue_, V_Red_ are respectively the volumes of contrast enhancement (CE), the blue and the red area of the tumor

* p<0.05


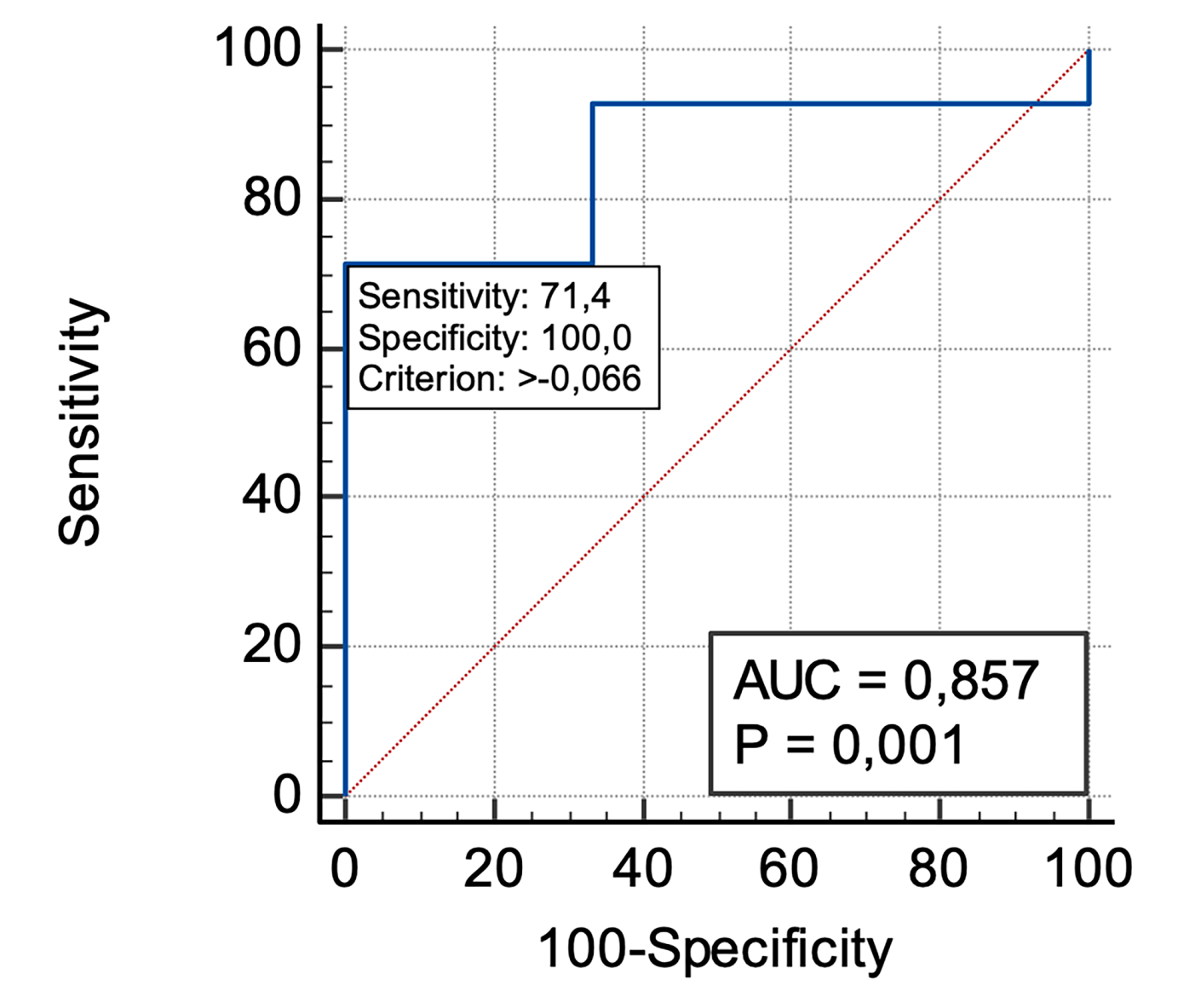


**Figure 1S**. ROC curve determining the optimal cut-off value to discriminate TTP (True Tumor Progression) and PsP (Pseudoprogression).


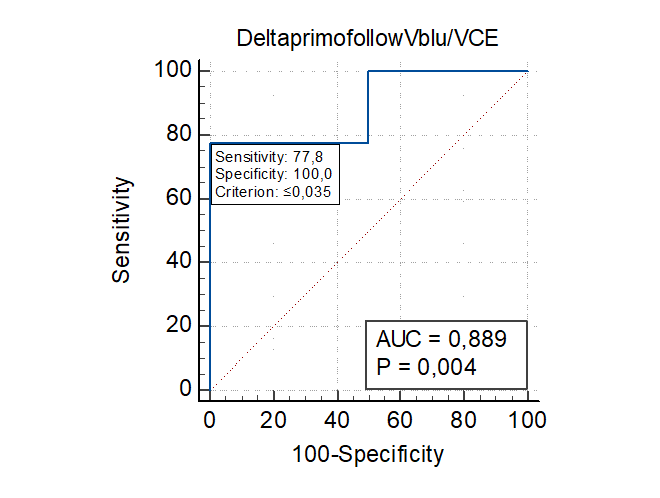


**Figure 2S**. ROC curve determining the optimal cut-off value to discriminate responder and non-responder patients at month 2.


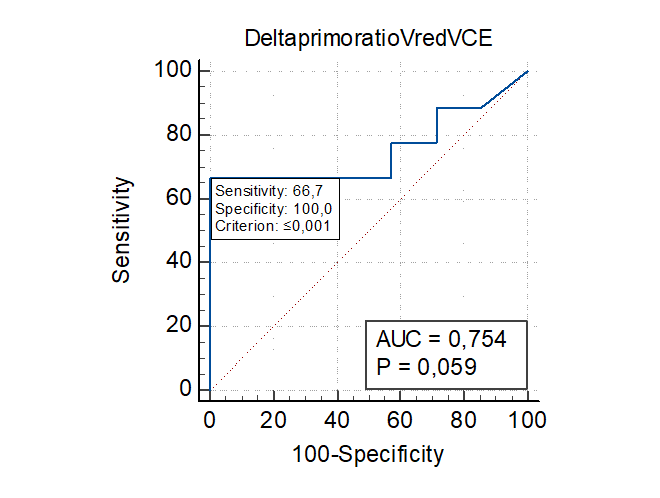


**Figure 3S.** ROC curve curve determining the optimal cut-off value to discriminate responder and non-responder patients at month 6.


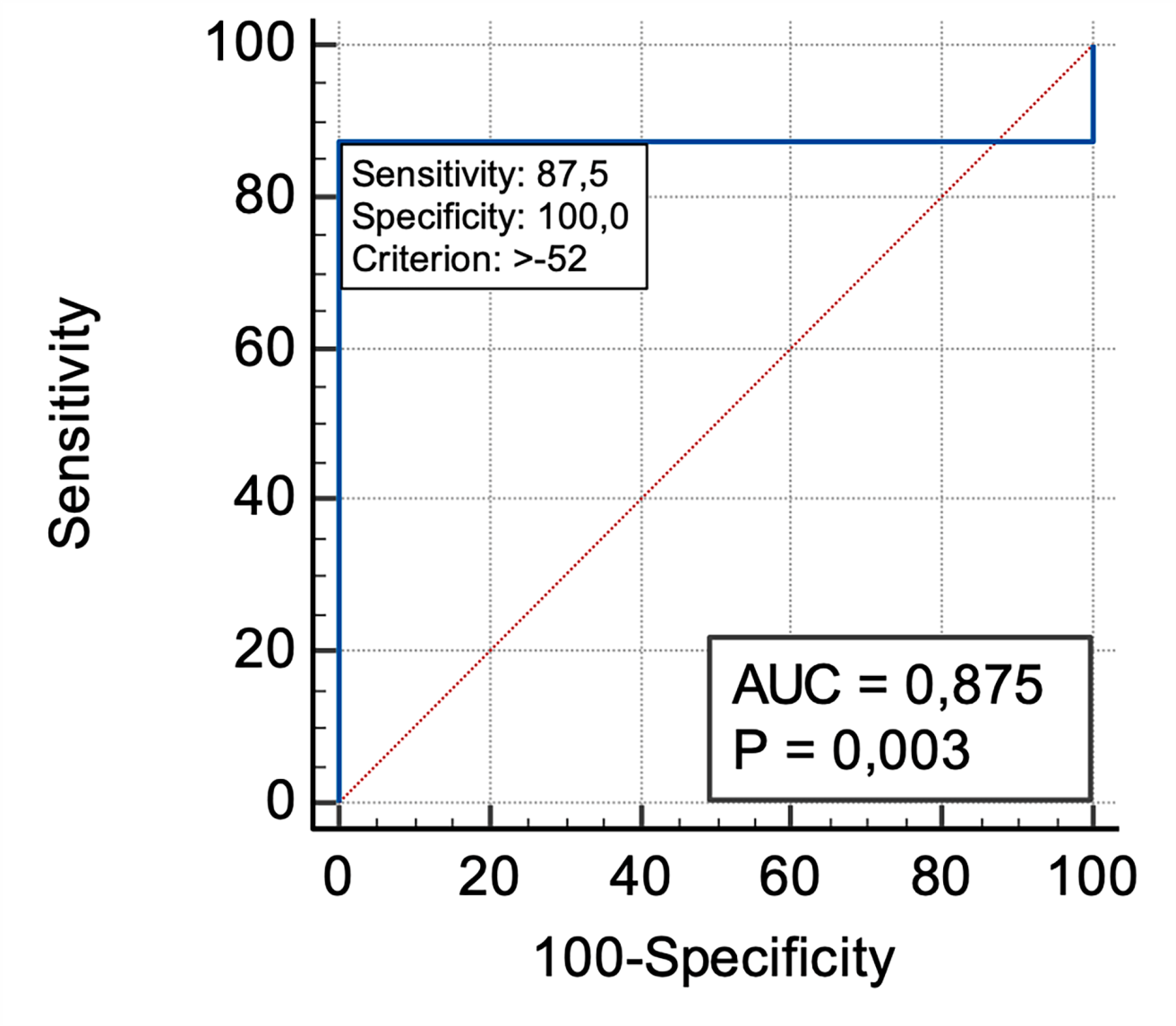


**Supplementary Figure 4S.** ROC curve determining the optimal cut-off value to discriminateHIGH-NK and LOW-NK patients
